# Consequences of insecticide resistance on malaria transmission

**DOI:** 10.1371/journal.ppat.1006499

**Published:** 2017-09-07

**Authors:** Haoues Alout, Benjamin Roche, Roch Kounbobr Dabiré, Anna Cohuet

**Affiliations:** 1 Institut des Sciences de l’Evolution de Montpellier, CNRS, IRD, University of Montpellier, Montpellier, France; 2 Institut de recherche pour le développement (IRD), Maladies Infectieuses et Vecteurs, Ecologie, Génétique, Evolution et Contrôle (MIVEGEC), Montpellier, France; 3 UMI IRD/UPMC 209, Unité de Modélisation Mathématique et Informatique des Sytèmes Complexes (UMMISCO lab), Bondy, France; 4 Institut de Recherche en Sciences de la Santé (IRSS), Bobo-Dioulasso, Burkina Faso; University of Wisconsin Medical School, UNITED STATES

## Malaria burden and control

Human malaria is caused by protozoan parasites of the genus *Plasmodium* (*Plasmodium falciparum*, *P*. *vivax*, *P*. *malariae*, *P*. *ovale*, and *P*. *knowlesi*). In 2015, 212 milion malaria cases were reported worldwide with approximately 429,000 deaths, 92% of which occured in Africa. However, several countries within sub-Saharan Africa have reported decreased numbers of malaria cases over the last several decades (http://www.who.int/malaria/publications/world-malaria-report-2016/en/). *Plasmodium* parasites are dependent on completing a complex life cycle in mosquitoes of the genus *Anopheles*, specifically, for transmission to occur. Thus, reducing the mosquito abundance or interfering with its ability to support the parasite cycle (i.e., the vector competence) can interrupt malaria transmission. However, not every species of *Anopheles* is a vector, and not all species of *Plasmodium* occur in all regions. In natural populations, the combination of *Plasmodium–Anopheles* species is very specific and leads to distinct epidemiology and transmission patterns. For example, the transmission of malaria parasites between humans is due primarly to the *A*. *gambiae s*.*l*.*–P*. *falciparum* combination, while in Southeast Asia, *A*. *dirus* and *A*. *minimus*–*P*. *vivax* are the main vectorial systems. This transmission potential between human hosts of a specific *Anopheles* population is characterized by the “vectorial capacity” and depends on the abundance, the feeding rate, the longevity, and the vector competence of female mosquitoes as well as the extrinsic incubation period of the parasite, which is the time for the pathogen to reach the transmissible stage from the midgut to the salivary glands of its vector. Between 2000 and 2015, the number of malaria infection deaths have been halved, with 79% of this reduction being attributed to insecticide-based vector control (insecticide-treated nets [ITNs] and indoor residual spraying [IRS]) [1]. Therefore, insecticide-based vector control still represents the most important and affordable method to alleviate the malaria burden by interrupting the transmission cycle, although insecticide-resistance frequency and intensity have increased dramatically in malaria vector populations. In an attempt to explain this discrepancy, we present 5 facts about insecticide resistance in regards to malaria control when the epidemiological and ecological contexts are considered.

## The threat of insecticide resistance

Only a few insecticides have been approved for public health, and, among them, only the pyrethroids are allowed for ITNs. Nowadays, resistance against this insecticide family is widespread among malaria vectors in Africa (see IR mapper [www.irmapper.com] for the latest update) and is expected to jeopardize the success of malaria control. According to the World Health Organization (http://www.who.int/malaria/publications/atoz/gpirm/en/), resistance to insecticides is defined as “an ability to tolerate doses of toxicants, which would prove lethal to the majority of individuals in a normal population of the same insect species”. Resistance arises from the selection of individuals able to survive and reproduce in an insecticide-treated environment or after being in contact with insecticides. Mechanisms that decrease the insecticide toxicity can rely on modifications in one or several genes of the mosquito, and, as a result, resistance is a heritable trait. In the presence of insecticides, the frequency of alleles responsible for insecticide resistance increases in the population because they confer a strong advantage, which is expected to hinder the effect of insecticides on vector survival and abundance.

The impact of insecticide resistance on vector control efficacy is usually measured by comparing survival rates of mosquito strains or natural populations exposed to various insecticides, both in the laboratory (e.g., WHO cones or tube assays) and in natural settings (e.g., experimental huts). The loss of insecticide efficacy may thus lead to partial operational failure of malaria control and probably to increased disease transmission. A meta-analysis of the published results synthetized the ITN-associated mortality of pyrethroid-resistant vectors and predicted a strong negative epidemiological impact of insecticide resistance [2].

## Discrepancy between entomological and epidemiological studies on insecticide-resistance impact

In contrast to these expectations based on entomological observations, implementation of vector control tools (ITNs and/or IRS) has significantly decreased malaria incidence and parasite infection prevalence in children in multiple endemic countries across Africa (Equatorial Guinea, Burundi, Ivory Coast, Malawi, and Kenya), despite moderate-to-high pyrethroid resistance observed in local malaria vectors [3–6]. These results show reductions in malaria indices following ITN distribution similar to those in areas with pyrethoid-susceptible vectors [4,7,8]. Only 1 study suggested that the selection of insecticide resistance has led to a rebound in malaria incidence in South Africa, but this could also be attributed to climatic factors, reestablishment of other malaria vector species, and resistance to antimalarial drugs [9]. There is thus no clear evidence that insecticide resistance would lead to malaria control failure; however, these studies were not specifically designed to test its epidemiogical impact and thus lack the power to assess it correctly. A trial in 5 countries (Benin, Cameroon, Kenya, Sudan, and India) comparing malaria incidence in areas of insecticide resistant versus susceptible vector populations is ongoing, and preliminary results did not show any evidence of an association between malaria burden and pyrethroid resistance (http://www.who.int/malaria/publications/atoz/insecticide-resistance-implications/en/), further supporting the previous observations that entomological efficacy of vector control does not directly correlate with epidemiological efficacy. In order to assess the actual threat of insecticide resistance on malaria control, it is fundamental to explain this contrast between the entomological and epidemiological outcomes of ITNs in insecticide-resistant vector populations.

## The phenotypic expression of insecticide resistance in malaria vectors

The detection of insecticide resistance in the field relies on standard protocols provided by WHO that use 1 insecticide dose, 1 exposure time, and the observation of mortality 24 h postexposure. However, the absence of mortality resulting from such methods does not imply a complete absence of mortality due to insecticides. For instance, the WHO protocols fail at capturing insecticide-induced delayed mortality, which is mortality occurring more than 24 h later [10]. In addition, the phenotypic expression of resistance and the resistance level are highly dependent upon environmental variables like temperature [11], food quality/quantity [12], multiple blood meals [13] and preexisting pesticide exposure [10], all variations that are not captured in standardized bioassays. Therefore, the standard protocols, while useful to monitor insecticide susceptibility during vector control campaigns, may not provide evidence for significant failure of disease control.

Similarly, the level of resistance has been shown to decline with age (reviewed in [14]), so that young mosquitoes found resistant could still die from exposure to insecticides when they get older. The fact that mosquitoes exposed to insecticide tend to die early-on in spite of their resistance alleles or the fact that they become more susceptible to insecticides when aging can have tremendous implications in terms of epidemiology: most of the infectious mosquitoes are indeed old females (*Plasmodium* requires about 10 days to become infectious once ingested by *Anopheles* vectors, which have a daily survivorship of approximately 0.8/day [15]), so insecticides may still be efficient at reducing their proportion and, thus, at controlling malaria transmission.

## The fitness cost of insecticide resistance and its impact on malaria transmission

The mutations responsible for resistance are often associated with modification of physiological processes or resource availability, which often leads to decreased performance and fitness disadvantage (i.e., cost). The deleterious pleiotropic effects (i.e., the negative influence on unrelated phenotypic traits) of insecticide-resistance alleles could affect a wide range of vector life-history traits, such as longevity, biting behavior, and vector competence [16], which are important factors of the vectorial capacity to transmit the pathogen. For instance, insecticide-resistance mutations have been shown to increase the vector competence of *A*. *gambiae* for malaria parasites [17], which may potentiate malaria transmission. The cost of insecticide resistance also depends on other biotic (i.e., parasite infection) and abiotic (i.e., environemental) factors, which may increase or decrease mosquitoes’ ability to transmit parasites. Parasite infection itself may impose an additional cost to vectors carrying insecticide resistance alleles, thereby further influencing the mosquito life-history traits involved in vectorial capacity and also the phenotypic expression of insecticide resistance. Consistently, infection by *Plasmodium* parasites was shown to alter the survival of resistant mosquitoes in the absence of insecticides [18] and to partially restore the susceptibility to insecticide in *A*. *gambiae* carrying the *kdr* mutation [19]. Moreover, exposure to pyrethroid insecticides reduced the prevalence of infection by *P*. *falciparum* in *A*. *gambiae s*.*s*. harboring the *kdr* mutation [20]. Altogether, these observations appear as assets for vector control. Therefore, the interactions between insecticide resistance, exposure, and infection induce contrasted effects on mosquito traits, so anticipating the “net” outcome of insecticide resistance on malaria epidemiology remains challenging.

## Conclusion

Considering only the direct effect of insecticide on mosquito survival often leads to the simplistic conclusion that insecticide resistance anihilates the efforts to control malaria incidence. There are at least 5 facts about the effect of insecticide resistance on malaria control: (1) the discrepancies between the entomological and epidemiological studies of malaria vector control efficacy in relation to insecticide resistance, (2) the overestimated phenotype of resistance in standard protocols compared to natural context (i.e., increased insecticide toxicity due to delayed insecticide effects or to age), (3) the fitness cost associated with insecticide resistance (on mosquito density, biting behavior, vector competence, and survival), (4) the increased parasite-induced mortality in insecticide-resistant mosquitoes (interactive cost between infection and resistance), and (5) the impact of insecticides on vector–parasite interactions (i.e., increased toxicity on infected/infectious vectors, reduced parasite development, and reduced transmission). In addition to these, other reasons for successful malaria control despite the selection of insecticide resistance exist, such as bed–net physical barrier and housing improvement (such as window screens and eave tubes). The local epidemiological, ecological, and entomological context (population structure, seasonality, multiple mechanisms of insecticide resistance, etc…) are thus crucial to consider to reach a more precise estimate of insecticide-resistance impact on vectorial capacity. We should also consider that continuous insecticide selective pressure, as well as parasitism, may select for enhanced resistance phenotypes and/or for reduced fitness cost. The epidemiological consequences of insecticide resistance are thus expected to be continuously moving. This emphasizes the necessity to precisely and continuously monitor the consequences of vector control strategies, considering the vectorial system in all its ecological interactions.
